# SCIA: A Novel Gene Set Analysis Applicable to Data With Different Characteristics

**DOI:** 10.3389/fgene.2019.00598

**Published:** 2019-06-25

**Authors:** Yiqun Li, Ying Wu, Xiaohan Zhang, Yunfan Bai, Luqman Muhammad Akthar, Xin Lu, Ming Shi, Jianxiang Zhao, Qinghua Jiang, Yu Li

**Affiliations:** ^1^Department of Laboratory of Cancer Biology, School of Life Science and Technology, Harbin Institute of Technology, Harbin, China; ^2^Department of Biostatistics, School of Public Health, Southern Medical University, Guangzhou, China

**Keywords:** GSA, competitive method, self-contained method, topology-based method, functional enrichment analysis

## Abstract

Gene set analysis is commonly used in functional enrichment and molecular pathway analyses. Most of the present methods are based on the competitive testing methods which assume each gene is independent of the others. However, the false discovery rates of competitive methods are amplified when they are applied to datasets with high inter-gene correlations. The self-contained testing methods could solve this problem, but there are other restrictions on data characteristics. Therefore, a statistically rigorous testing method applicable to different datasets with various complex characteristics is needed to obtain unbiased and comparable results. We propose a self-contained and competitive incorporated analysis (SCIA) to alleviate the bias caused by the limited application scope of existing gene set analysis methods. This is accomplished through a novel permutation strategy using *a priori* biological networks to selectively permute gene labels with different probabilities. In simulation studies, SCIA was compared with four representative analysis methods (GSEA, CAMERA, ROAST, and NES), and produced the best performance in both false discovery rate and sensitivity under most conditions with different parameter settings. Further, the KEGG pathway analysis on two real datasets of lung cancer showed that the results found by SCIA in both of the two datasets are much more than that of GSEA and most of them could be supported by literature. Overall, SCIA promisingly offers researchers more reliable and comparable results with different datasets.

## Introduction

In recent years, gene set analysis (GSA) has become the most common method in functional genomics studies, because evaluating a single *p*-value for a gene set is statistically more powerful than genewise tests. Typically, by choosing gene sets that represent biological pathways, GSA can help to bring insights into biological mechanisms, cellular functions, and disease states (Kanehisa et al., 2012). Various statistical procedures for gene set testing have been proposed and can be divided into three generations roughly in chronological order (Khatri et al., 2012; Zyla et al., 2017). The first generation of GSA used over-representation analysis (ORA), where the first step is to define differentially expressed genes (DEGs) and non-DEGs in the input gene list by a certain threshold (Beissbarth and Speed, 2004). Then, the proportion of DEGs between a given functional gene set and the background gene set are tested by hypergeometric, binomial, or chi-square distribution. This comparison of the DEG proportions is the original theory of competitive testing. ORA has been reported with minor variations by many different authors (Khatri and Draghici, 2005). Even though the ORA method seems simple and effective, there are two serious drawbacks. First, the information about the strength of gene expression is lost by gene binarization. Second, the assumption of inter-gene independence needed by the testing methods is not satisfied in most cases.

The second generation of GSA, known as functional class sorting (FCS), was proposed to avoid these deficiencies. Instead of defining genes as DEGs and non-DEGs, different univariate gene-level statistics such as *t*-statistic (Al-Shahrour et al., 2005; Tian et al., 2005), *Q*-statistic (Goeman et al., 2004), signal-to-noise ratio (Subramanian et al., 2005), fold change score and *Z*-score (Kim and Volsky, 2005), or their trans-formations (Tian et al., 2005; Ackermann and Strimmer, 2009) are used to measure DEGs and overcome the first problem of ORA. Then, a gene-set-level statistic is aggregated by these gene-level statistics. Aggregation approaches can be sum, mean, median of the gene-level statistics (Jiang and Gentleman, 2007), or calculating statistics such as the Kolmogorov-Smirnov statistic (Mootha et al., 2003; Subramanian et al., 2005), Wilcoxon rank sum (Barry et al., 2005), or the max-mean statistic (Efron and Tibshirani, 2006). Because the distributions of gene-set-level statistics are usually unknown, permutation procedures are used to complete FCS tests. According to different null hypotheses and corresponding permutation objects, FCSs can be classified as competitive or self-contained methods.

Assuming that all the input genes are independent of each other, competitive methods usually permute gene labels but lose the inter-gene information, which causes the false discovery rate (FDR) to be uncontrolled when the inter-gene correlations are high. Self-contained methods test each gene set independently by permuting sample labels but lose all the information outside the given gene set, which causes the FDR to be uncontrolled when the percentage of DEGs in the background genes is high. Irrespective of the prerequisites for the permutation procedure, the ORA methods can be considered as generalized competitive methods, whereas the classical methods based on multiple linear regression (Mansmann and Meister, 2005; Kong et al., 2006), by definition, are special cases of self-contained methods.

To address the second problem of ORA, some competitive FCS methods that take account of the correlations among genes have been proposed. The method of Nam (2010) removed the bias caused by the inter-gene correlations, while the method of Wu and Smyth (2012) alleviated the problem by estimating the variance inflation factor. However, the information of inter-gene correlations is partially neglected in these procedures, which causes reduced sensitivity or uncontrolled FDR. Self-contained FCS methods seem to be more powerful than competitive ones and do not assume that all the genes are independent, but their null hypothesis is usually over restrictive (Goeman et al., 2004; Tian et al., 2005; Khatri et al., 2012). They assume that the gene set does not contain any genes with expression levels that are associated with different experimental conditions. Under this hypothesis, a few DEGs may cause a given pathway to be defined as a significant differential pathway (Khatri et al., 2012). Although the method of Wu et al. (2010) moderated this hypothesis using a Monte Carlo based testing method, the parameter describing the least proportion of DEGs in a pathway is given arbitrarily instead of calculated by the expression of genes outside the gene set. Even though competitive methods are overwhelmingly more commonly used than self-contained methods in the genomic literature (Gatti et al., 2010), information is still lost during the permutation procedures. Thus, the collision of applicable scopes between self-contained and competitive methods remains unsolved.

The third generation of GSA, known as the pathway topology (PT)-based approach, is based on the large amount of publicly available pathway knowledge. Mitrea et al. (2013) introduced dozens of PT-based methods with different principles and applicable conditions. Most of these methods consider topological information as a weight that measures the centrality of nodes but ignores the spatiotemporal specificity of topological information and changes in the topological structure between different experimental conditions (Fang et al., 2012; Gu et al., 2012; Dona et al., 2017). On this basis, the method of Yuan et al. (2016) proposed a novel statistic that combines node (gene expression) changes with edge (inter-gene correlation) changes. The utilization of biological information greatly improved the performance of PT-based methods, however, the testing methods of them are essentially the same as FCS methods in that they perform the same pipeline (Mitrea et al., 2013). Therefore, the above defects of FCS methods are not solved by PT-based methods.

Here, we propose a new GSA method with less information loss that can alleviate the bias of self-contained and competitive methods caused by their limited applicability. First, to capture all the information within a given gene set like other self-contained methods, a powerful multivariate statistic C is developed to test node changes and edge changes simultaneously. We chose Hotelling's *T*^2^, a self-contained statistic with the ability to penalize gene collinearity (Ackermann and Strimmer, 2009), for node testing because of its suitability for overcoming the limitation of competitive methods, and linear regression to test the edge changes among genes. Because of the additivity of chi-square distributed variables, these two statistics are transformed to the chi-square scale and summed up to get the C statistic. Second, we developed a novel permutation procedure based on a condition-specific shortest-path network (CSSPN, proposed by Dezso et al., 2009). The genes in the CSSPN are selectively permuted instead of permuting the whole gene labels as usual. This procedure does not disrupt inter-gene correlations but uses inter-pathway information from *a priori* biological networks, which creates a platform for the incorporation of self-contained, competitive, and PT-based methods. The whole pipeline is called self-contained and competitive incorporated analysis (SCIA), which has been implemented in an R package “SCIA” available on GitHub https://github.com/YiqunLiHIT/SCIA. Results from this study showed that the sensitivity and FDR of SCIA outperform four other commonly used GSA methods in most conditions in simulated datasets and the results are more stable with different real datasets of lung cancer.

## Statistical Models and Methods

### Notations and Background Network

The main objective of SCIA is to detect gene sets that are differentially expressed under different experimental conditions. Here, we consider the gene set as pathway *P* for one experimental condition and *P*′ for another. N_1_ and N_2_ are the sample size for *P* and *P*′, respectively. For convenience, we assumed that *P* and *P*′ are under linear models:

$$\begin{array}{l} X_{1}\overset{\beta_{1}}{\to }X_{2}\overset{\beta_{2}}{\to }\ldots \ldots \;X_{n-1}\overset{\beta_{n-1}}{\to }X_{n}\\ {X^{\prime }}_{1}\overset{\beta_{1}{}^{\prime }}{\to }X_{2}{}^{\prime }\overset{\beta_{2}{}^{\prime }}{\to }\ldots \ldots \;{X^{\prime }}_{n-1}\overset{{\beta^{\prime }}_{n-1}}{\to }{X^{\prime }}_{n} \end{array}$$

with *n* nodes and *n* − 1 edges, where β_*i*_ (1 ≤ *i* < *n*) represent the regression coefficient of *X*_*i*_ and *X*_*i*+1_. Let *U* = $\left({\bar{X}}_{1}-\;{\bar{X}}_{1}{}^{\prime },{\bar{X}}_{2}-\;{\bar{X}}_{2}{}^{\prime },\;\ldots \ldots ,\;{\bar{X}}_{n}-\;{\bar{X}}_{n}{}^{\prime }\right)$ denote the vector of difference in the means of two groups. *S* and *S*′ are the covariance matrices of *P* and *P*′, respectively. These notations are also used in the simulation studies.

We chose the background network of CSSPN as the Human Protein Reference Database (HPRD) network (Library et al., 2009), a centralized platform to visually depict and integrate information pertaining to do-main architecture, post-translational modifications, interaction networks, and disease associations for each protein in the human proteome. Other comprehensive networks, such as the integrated network of seven common used networks in Edge Set Enrichment Analysis (Han et al., 2015) can also be used as the background network of SCIA.

### *C* Statistic

The *C* statistic is proposed to measure the difference of a given gene set in different experimental conditions. It consists of two parts, the node difference model and the edge difference model. The node difference model is based on Hotelling's *T*^2^ method:

$$\begin{array}{r} T^{2}\;=\frac{N_{1}N_{2}}{N_{1}\;+\;N_{2}}U^{T}S_{c}^{-1}U \end{array}$$

where,

$$\begin{array}{r} S_{c}=\;\frac{\left(N_{1}-1\right)S\;+\;\left(N_{2}-1\right)S^{\prime }}{N_{1}\;+\;N_{2}-2} \end{array}$$

Under the self-contained null hypothesis *H*_0_: *U* = 0, *T*^2^ follows a chi-square distribution with degrees of freedom equal to *n* representing genes in the given pathway with a sufficient sample size. This allows Hotelling's *T*^2^ statistic to be combined with other statistics that also follow a chi-square distribution, because chi-square distributions are additive on the freedoms. There are many transformations of Hotelling's *T*^2^ statistic which show its different characteristics. It can be transformed as:

$$\begin{array}{r} F=\;\frac{N_{1}\;+\;N_{2}-n-1}{\left(N_{1}\;+\;N_{2}-2\right)n}T^{2} \end{array}$$

following an *F* distribution with the degree of freedom of *n* and *N*_1_ + *N*_2_ − *n* − 1 under a relatively small sample size. This allows Hotelling's *T*^2^ statistic to be used alone when the sample size is insufficient. Typically, Hotelling's *T*^2^ test is not only a node testing method but is related to the Pearson correlation coefficient. For convenience, assuming *n* = 2 and *N*_2_ is big enough, the estimated value ${{\bar{X}}_{i}}^{\prime }\;$(1 ≤ *i* ≤ 2) can be considered as constants μ_*i*_ (1 ≤ *i* ≤ 2), then Hotelling's *T*^2^ statistic can be transformed as:

$$\begin{array}{r} T^{2}\;=\;\frac{t_{1}^{2}+t_{2}^{2}-2\rho t_{1}t_{2}}{1-\rho^{2}} \end{array}$$

where *t*_1_ *and t*_2_ denote the *t-*statistics for the two component genes, and ρ represents the Pearson correlation coefficient between *X*_1_ and *X*_2_. If *t*_1_ = *t*_2_, Hotelling′s *T*^2^ statistic can be simplified to:

$$\begin{array}{r} T^{2}\;=\frac{2t_{1}^{2}}{1+\rho } \end{array}$$

This transformation of *T*^2^ indicates that when *X*_1_ and *X*_2_ are positively correlated and have similar changes in different experimental conditions, there would be a penalty on the Pearson correlation coefficient, which can avoid the disadvantages of the competitive methods. When *X*_1_ and *X*_2_ are negatively correlated but both have positive changes in different experimental conditions, which indicates that the correlation of *X*_1_ and *X*_2_ has changed in different experimental conditions, the *T*^2^ statistic is would be more sensitive.

Although Hotelling's *T*^2^statistic only slightly considers the correlations between genes, a statistically rigorous edge testing statistic is still needed. Based on the linear regression method, a *Z*-score-like statistic is combined with Hotelling's *T*^2^ statistic in the *C* statistic. ${\hat{\beta }}_{i}\;$and ${\hat{\beta }}_{i}{}^{\prime }$ can be estimated by the least square method. Then the *Z*-score-like *B* statistic can be written as:

$$B_{i}=\frac{{\hat{\beta }}_{i}-{\hat{\beta }}_{i}{}^{\prime }}{\sqrt{var\left({\hat{\beta }}_{i}\right)+var\left({\hat{\beta }}_{i}{}^{\prime }\right)}}$$

under the null hypothesis *H*_0_: ${\hat{\beta }}_{i}$ = ${\hat{\beta }}_{i}{}^{\prime }$, *B*_*i*_ follows a standard normal distribution the same as the *Z*-score, and ${\;B}_{i}^{2}\;$follows a chi-square distribution and can be combined with Hotelling's *T*^2^ statistic. Thus, we obtained the *C* statistic as:

$$\begin{array}{r} C=T^{2}+\;\overset{n-1}{\underset{i=1}{\sum }}{\text{B}}_{i}^{2} \end{array}$$

which follows a chi-square distribution with the degrees of freedom equal to *n*+(*n*−1), and can be used to test node changes and edge changes simultaneously. Notably, when the sample size is very small, *T*^2^ and ${\text{B}}_{\text{i}}^{2}$ will not obey the chi-square distribution, the parameter of SCIA about the correlation test should be set as “FALSE.”

### CSSPN-Based Permutation Procedure

To avoid the shortcoming of self-contained methods and utilize additional inter-pathway information from *a priori* biological networks, a CSSPN is built by SCIA. First, a set of DEGs should be selected as the terminal genes of CSSPN, and a set of initial genes can usually be selected in the same way. For each pair of genes (*X*_*i*_, *X*_*t*_), where *X*_*i*_ is in the initial gene set and *X*_*t*_ is in the terminal gene set, all the shortest pathways are searched under a background network, such as HPRD (see section Notations and Background Network). When the results are not unique, the pathway with the highest *C* score will be chosen for a sub-pathway permutation procedure. In this procedure, 1,000 nodes are selected randomly as the initial gene set for each *X*_*t*_, which is the only terminal gene in this procedure. Assuming there are *x* shortest pathways, built by the randomly selected genes and *X*_*t*_, that have higher *C* scores than the given gene pair (*X*_*i*_, *X*_*t*_), the permutation *p*-value of the sub-pathway (*X*_*i*_, *X*_*t*_) is *x*/1,000. The permutation *p*-value and *C* statistic *p*-value are both adjusted using the method of Benjamini and Hochberg (1995), and only if the two *p*-values are <0.05, the sub-pathway is defined as a statistically significant pathway. Then, all the significant sub-pathways among the initial gene set and the terminal gene set are used to build the CSSPN. All the genes in the CSSPN can be considered as DEGs with edges and can be used in classical functional enrichment analysis.

In SCIA, background genes are used selectively in the CSSPN-based permutation procedure. Essentially, the selection of background genes means the information from the *a priori* biological network is utilized, because all the genes neighboring DEGs in the background network are used at a higher probability to establish the CSSPN. Additionally, because the permutation procedure does not destroy any inter-gene or inter-pathway structures, almost no information is lost in SCIA.

## Results

### Simulated Data and Scenarios

#### Simulated Data

The simulated data were generated under a linear model (Formula 1). Firstly, we generated the initial node *X*_1_ of a given pathway *P* from the normal distribution $N\;\left(\mu_{1},\;{\sigma_{1}}^{2}\right)$. And then, the neighbor node *X*_2_ = β_1_*X*_1_ + ε_1_, *X*_3_ = β_2_*X*_2_ + ε_2_ …… *X*_*n*_ = β_*n*−1_*X*_*n*−1_ + ε_*n*_ were generated in the same way. Where $\varepsilon_{i}\;\sim \;N\left(0,{\tau_{i}}^{2}\right)$(1 < *i* ≤ *n*) was the residual error term. Similarly, we generated $X_{1}{}^{\prime }\;\sim \;N\left(\mu_{1}{}^{\prime },\sigma_{1}{{}^{\prime }}^{2}\right),X_{1}{}^{\prime }=\beta_{i-1}{}^{\prime }X_{i-1}{}^{\prime }+\varepsilon_{i}{}^{\prime }$ with $\varepsilon_{i}{}^{\prime }\;\sim \;N\left(0,\;\tau_{i}{{}^{\prime }}^{2}\right)\;(1<i\leq n)$representing the pathway *P*′under another experimental condition. Under the *H*_0_ hypothesis that there is no change in nodes and edges between different experimental conditions, we set the default simulating parameters as: $\mu_{1}=\mu_{1}{}^{\prime }=\;1,\;\sigma_{1}{}^{2}\;=\;\sigma_{1}{{}^{\prime }}^{2}=1,\;\tau_{i}{}^{2}=\;\tau_{i}{{}^{\prime }}^{2}=1,\text{ and }\beta_{i}=\;\beta_{i}{}^{\prime }=0.5$. In most of the following simulations without mentioned specially, the gene number *n* in a pathway was set as 5, the sample sizes *N*_1_ and *N*_2_ of different experimental conditions were both set as 100, and the simulations were repeated 1,000 times.

#### Scenarios

Four scenarios and 16 conditions were used to simulate different data structures and prove the extensive applicability of SCIA. The *H*_0_ hypothesis condition was designed to evaluate the FDR and the *H*_1_ hypothesis condition was designed to evaluate the sensitivity. The basic setting for the *H*_1_ hypothesis is node or edge changes, with three additional conditions: sample size, inter-gene correlation, and percentages of DEGs in background genes that are outside the given pathway. In each scenario, only one additional condition is set as different values to highlight the robustness of SCIA. Thus, the four scenarios are:

1. Node change, 0% background DEGs, different correlations, and fixed sample size.
2. Node change, 10% background DEGs, different correlations, and fixed sample size.
3. Node change, different percentages of background DEGs, fixed correlations, and fixed sample size.
4. Edge change, 0% background DEGs, fixed correlations, and different sample sizes.

Scenarios 1 and 2 were designed to simulate datasets with different inter-gene correlations, scenario 3 was designed to simulate datasets with different percentages of DEGs in background genes, and scenario 4 was designed to simulate datasets with edge changes under different sample sizes. Details of the parameter settings under these scenarios are listed in Supplementary Data Section 1.

## Evaluation of SCIA Performance With Simulated Data

To evaluate its performance, SCIA was compared with two powerful self-contained approaches, ROAST and NES, and two commonly used competitive approaches, CAMERA and GSEA (More details about these methods are stated in Supplementary Data Section 2). The application scope of these methods is quite different, so we compared SCIA with them under corresponding application conditions. As shown in Table 1, only competitive methods are suitable for scenario 3, and only self-contained methods are suitable for scenario 4.

**Table 1:** Application scope of the different methods evaluated in this study.

**Conditions**	**SCIA**	**Self-contained**		**Competitive**	
		**NES**	**ROAST**	**CAMERA**	**GSEA**
High intergene correlations	√	√	√	√	×
High prop. of background DEGs	√	×	×	√	√
Correlation changes testing	√	√	×	×	×

*“√” indicates the method was designed for the condition; “ × ” indicates the method was not designed for the condition and may have problems in sensitivity or FDR*.

### SCIA Successfully Controls the FDR Under Different Inter-gene Correlations in Simulated Datasets

**Figure 1:**
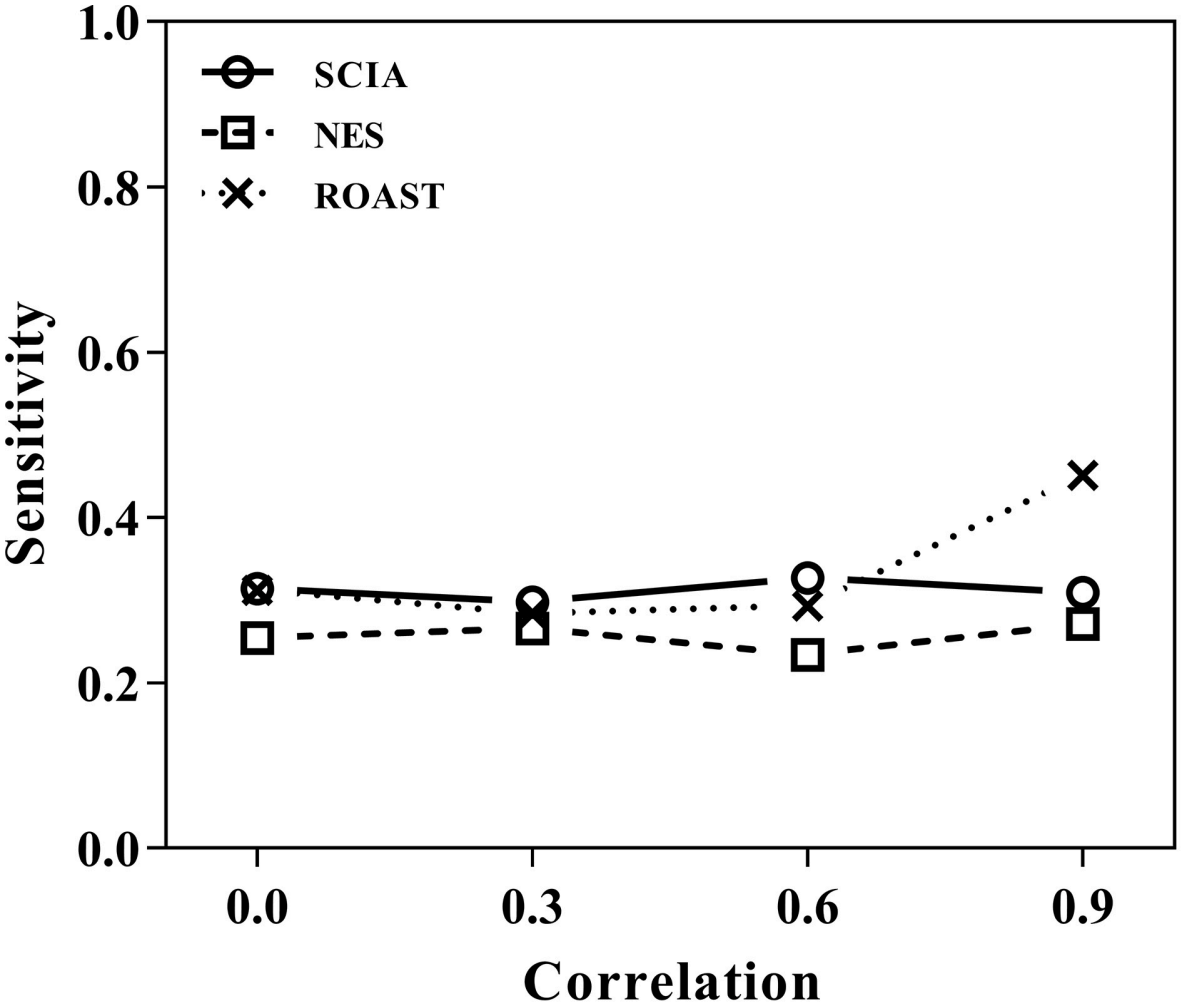
SCIA produces sensitivities similar to those for the self-contained methods under different inter-gene correlations in simulated datasets.

First, we compared SCIA with self-contained methods in scenario 1 under different inter-gene correlations in simulated datasets. The FDRs were well-controlled by all the three methods (Table 2), and Figure 1 clearly shows the sensitivities of the three methods were quite similar, indicating the *C* statistic allowed SCIA to match the advantages of the self-contained methods. Noticeably, ROAST had high sensitivity under the high inter-gene correlation. However, high sensitivity with inter-gene correlations close to 1 is not useful for combination with competitive approaches because a small percentage of highly correlated DEGs may produce unreasonable significant results.

**Table 2:** FDR is well-controlled by SCIA similar to other self-contained methods under different inter-gene correlations in simulated datasets.

**Correlations**	**SCIA**	**NES**	**ROAST**
0.0	0.048	0.056	0.052
0.3	0.046	0.045	0.046
0.6	0.056	0.049	0.061
0.9	0.044	0.082	0.038

Second, we compared SCIA with competitive methods under scenario 2. Table 3 clearly shows that the FDR of GSEA lost control, which is common for competitive methods due to the correlation between genes, whereas CAMERA adjusted the high FDR only under a moderate inter-gene correlation of all genes but failed to control the FDRs under high inter-gene correlations. SCIA was the most robust method with well-controlled FDRs and similar sensitivities as CAMERA with comparable FDRs. Because there were no randomly selected DEGs in the given pathway, the SCIA results in scenarios 1 and 2 are comparable, which indicated that the information of background genes outside the given gene set was well-utilized by SCIA. A notable question is that the intersection ratio of the results obtained from SCIA and GSEA is decreasing with the increasing of inter-gene correlation, because GSEA is more sensitive in finding significant pathways with less but consistent expression changes. This result indicated that SCIA and GSEA could find different types of differentially expressed gene sets.

**Table 3:** SCIA has lower FDRs than the competitive methods under different inter-gene correlations in simulated datasets.

**Pearson correlation coefficients**	**FDR**			**Sensitivity**		
	**SCIA**	**CAMERA**	**GSEA**	**SCIA**	**CAMERA**	**GSEA**
0.0	0.016	0.048	0.042	0.286	0.183	0.126
0.3	0.018	0.065	0.112	0.257	0.287	0.304
0.6	0.029	0.104	0.216	0.256	0.442	0.529
0.9	0.033	0.381	0.424	0.304	0.821	0.297

### SCIA Has Higher Sensitivity and Lower FDR Than Two Competitive Methods Under High Percentages of DEGs in Background Genes

When the percentages of DEGs in background genes are high, there are likely to be relatively high overlaps between a given gene set and background DEGs. Therefore, self-contained methods are invalid in scenario 3 and SCIA was compared with competitive methods. Table 4 shows that SCIA had higher sensitivity than the other two methods and, interestingly, the FDR was negatively correlated with the percentage of DEGs in background genes. These results are reasonable and reflect the incorporation of different GSA methods in SCIA. Like other competitive methods, when the percentage of DEGs in background genes was high, SCIA assigned a competitive penalty of the significance to the given pathway, and when the percentage of DEGs in background genes was low, SCIA assumed only a few percentages of the DEGs would produce a significant result for the given pathway because there was no other explanation for these DEGs. Notably, in complex diseases such as cancer, DEGs usually account for more than 40% of the genes in a dataset, under which condition SCIA was the best method both in sensitivity and FDR.

**Table 4:** SCIA has higher sensitivity than the competitive methods under different percentages of DEGs in background genes.

**Proportion**	**FDR**			**Sensitivity**		
	**SCIA**	**CAMERA**	**GSEA**	**SCIA**	**CAMERA**	**GSEA**
0.2	0.157	0.124	0.188	0.760	0.580	0.507
0.4	0.112	0.138	0.161	0.788	0.559	0.513
0.6	0.093	0.151	0.169	0.816	0.528	0.413

### SCIA Has Higher Sensitivity Than the Two Self-Contained Methods in Testing Changes of Inter-gene Correlations

**Figure 2:**
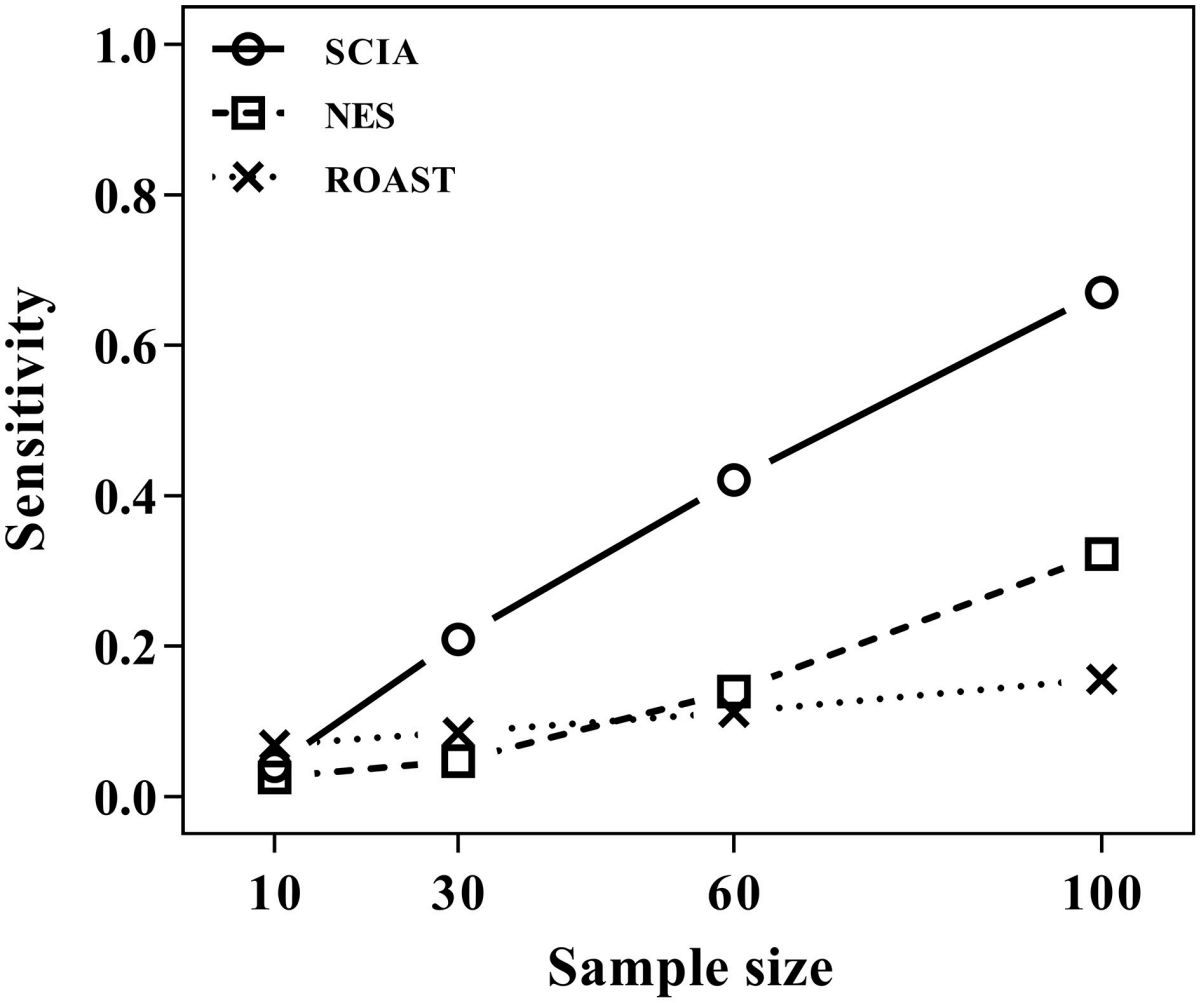
SCIA performs better in edge testing than the self-contained methods under different inter-gene correlations and different sample sizes.

Most competitive methods cannot simultaneously test node and edge changes; hence, we compared SCIA with self-contained methods under scenario 4 with the same *H*_0_ hypothesis and FDRs (Table 2) as scenario 1. The influence of different sample sizes was measured at the same time. Figure 2 shows that SCIA had the highest sensitivity and the slowest drop in sensitivity with decreasing sample sizes. However, when the sample size was 10 pairs, the sensitivity of SCIA dropped sharply because of the approximation of chi-square distribution (see method), which needs sample sizes of 15–30 pairs. Unsurprisingly, ROAST had the lowest sensitivity because it was not designed for this purpose. Besides, although the edge testing modules of SCIA and NES are quite similar, SCIA was more sensitive because edge changes are also considered by Hotelling's *T*^2^ (see method), indicating SCIA does not simply superpose node testing and edge testing methods like NES.

## Evaluation of SCIA Performance With Real Datasets

We applied SCIA to recover differentially expressed genes and pathways involved in lung squamous cell carcinoma (LUSC), a common type of non-small-cell lung cancer using two datasets, one from the NCBI's GEO (Gene Expression Omnibus) and one from TCGA (The Cancer Genome Atlas) database. The GEO dataset (Series Accession: GSE103512, Brouwer-Visser et al., 2017) contains 23 LUSC sub-type cancer samples and 9 normal samples. The LUSC dataset from TCGA contains 502 LUSC samples and 51 normal samples.

The two LUSC datasets were used as input to compare the sensitivity and robustness of SCIA and GSEA. In the CSSPN-base permutation procedure of SCIA, all the genes were mapped to the HPRD network, then the top 2% of DEGs (about 200 in each dataset) were defined as the initial and terminal genes of CSSPN (see method). All the nodes in the CSSPN were used for classical functional enrichment analysis based on a hypergeometric test. Unlike the simulation studies, the adjustment of permutation *p*-values (see method) should be moderate here. This is because, under the *H*_0_ hypothesis of simulation studies, there is no relation between the background network and the given gene set, whereas, in real organisms, hundreds of genes in the background network will differentially expressed in response to the DEGs in the given gene set. Due to the *C* statistic *p*-values of all the single pathways were already Benjamini and Hochberg (1995) adjusted, we did not adjust the permutation *p*-value in the following analysis, indicating there are approximate 500 genes in the HPRD background network that, on average, are affected by the terminal DEGs. This *p*-value threshold is a parameter of SCIA and can be set as different scores according to different data and requirements.

The results of the KEGG functional enrichment analysis are shown in Supplementary Tables S1–S4. SCIA found 131 and 64 pathways and GSEA found 46 and 40 pathways in the GSE103512 and TCGA LUSC datasets, respectively. Among them, 55 (42%) SCIA pathways were common between the two datasets, whereas only 5 (11%) of the GSEA pathways were common between the two datasets. These results illustrated that there was little comparability between the two results of GSEA, while, SCIA could demonstrate common results in different lung cancer datasets and the individual differences in the two researches, implying the two results of SCIA with different datasets were comparable. More than 33 of the 55 SCIA pathways found in both of the two datasets have been reported previously to have relationships with lung cancer (Table 5), including the non-small cell lung cancer. While, most of these pathways were not detected by GSEA. This result showed that SCIA could find many positive pathways that GSEA could not, and the high proportion of results with literature supporting indicated that the intersection of results of SCIA with different datasets could increase the reliability. Further, SCIA produces a CSSPN, which can be considered simply as a set of DEGs. SCIA detected 41 DEGs in the two datasets, and more than 27 (Supplementary Table S5) of these genes have been reported previously to be related with lung cancer.

**Table 5:** SCIA found more literature supported KEGG pathways than GSEA in two non-small-cell lung cancer datasets.

**KEGG pathway name**	**Adjusted *p*-value of SCIA**	**GSEA**
Cell cycle	3.89E-45	Yes
Cellular senescence	3.99E-12	No
Epstein-Barr virus infection	2.31E-11	Yes
Viral carcinogenesis	5.59E-10	Yes
p53 signaling pathway	4.81E-09	Yes
FoxO signaling pathway	1.19E-08	No
Platinum drug resistance	2.16E-07	Yes
Hepatitis B	1.43E-06	No
Transcriptional misregulation in cancer	1.92E-06	No
Small cell lung cancer	5.74E-06	No
Human papillomavirus infection	1.39E-05	No
MicroRNAs in cancer	1.62E-05	No
Glioma	3.25E-05	No
Kaposi's sarcoma-associated herpesvirus infection	3.10E-05	Yes
Apoptosis	3.51E-05	No
Non-small cell lung cancer	5.11E-05	No
Hepatocellular carcinoma	9.52E-05	No
Hippo signaling pathway	0.0001275	No
TGF-beta signaling pathway	0.0004040	No
Adherens junction	0.0006536	No
PI3K-Akt signaling pathway	0.0006624	No
Proteoglycans in cancer	0.0058405	No
Wnt signaling pathway	0.0084030	No
AGE-RAGE signaling pathway in diabetic complications	0.0151588	No
HIF-1 signaling pathway	0.0302121	No
Hepatitis C	0.0339220	No
Basal cell carcinoma	0.0343406	No
Mitophagy—animal	0.0362401	No
ErbB signaling pathway	0.0418948	No
Insulin resistance	0.0418948	No
Apoptosis—multiple species	0.0427196	No
Measles	0.0427196	No
Amyotrophic lateral sclerosis (ALS)	0.0427196	No

“*Yes” means the pathway is found by both SCIA and GSEA with adjusted p-value <0.05. “No” means the pathway is found by SCIA but not by GSEA*.

## Discussion

SCIA is the first GSA method that combines the advantages of self-contained, competitive, and PT-based methods. SCIA has three main advantages over the other methods as was shown by the simulation studies. First, SCIA is powerful and statistically rigorous under high inter-gene correlations, which are conditions under which most competitive methods lose control of FDR. Second, SCIA has higher sensitivity and minimum FDR compared to two competitive methods (GSEA, CAMERA) under a high proportion of DEGs in background genes, which are conditions that make most self-contained methods invalid. Moreover, SCIA uses an *a priori* biological network and performs better than ROAST and NES in testing edge (inter-gene correlation) changes. Overall, the FDR of SCIA was well-controlled and its sensitivity was higher than that of the other four methods tested (GSEA, CAMERA, ROAST, and NES) under most simulated conditions, highlighting the extensive applicability and unbiased results of SCIA.

The robustness of SCIA can be attributed to two aspects. First, its extensive applicability with reliable and unbiased results, as mentioned above, are the most important reasons. Second, through the CSSPN-based permutation strategy in SCIA, a reasonable hypothesis is innovatively combined with *a priori* biological information. Briefly, if DEGs can be mapped only in one gene set, a positive weight is added to them because there is no other explanation for the differential expressions of these genes. Therefore, for SCIA, comprehensiveness of the background networks is more important than its accuracy. However, when the *a priori* biological networks are more comprehensive, the hypothesis of SCIA becomes more reasonable and the results are more precise. This robustness gives SCIA the ability to calculate with different datasets and to integrate the results of SCIA with different datasets.

There are many potential applications for SCIA, including differential expression analysis (Dona et al., 2017), sub-pathway analysis (Martini et al., 2013), and micorRNA target gene prediction (Wang, 2008). First, all of the genes in the CSSPN can be considered as DEGs and used independently. In addition, CSSPN itself can be considered as a cascading effect pathway when the input data are from a knockout/over-expression experiment of a single gene. Second, if the function of differential pathways can be biologically confirmed, the sub-pathway of the given functional pathway can be built without the permutation procedure. Third, the choice of initial gene set is very flexible and can be tailored for different purposes. For instance, if the input data are derived from a microRNA knockout/over-expression experiment, the initial gene set can be select as the predicted target genes of the microRNAs, and the significant predicted targets will have more potential to be the targets of these microRNA in a specific experimental condition.

## Data Availability

Publicly available datasets were analyzed in this study. This data can be found here: https://www.ncbi.nlm.nih.gov/geo/query/acc.cgi?acc=GSE103512; https://www.cancer.gov/about-nci/organization/ccg/research/structural-genomics/tcga.

## Author Contributions

YuL and QJ designed the experiments. YiL, YW, YB, XZ, and JZ performed the experiments and data analysis. LA, XL, and MS have contributed to the writing of this article.

## Conflict of Interest Statement

The authors declare that the research was conducted in the absence of any commercial or financial relationships that could be construed as a potential conflict of interest.

## Abbreviations

GSA: gene set analysis
ORA: over-representation analysis
DEGs: differentially expressed genes
FCS: functional class sorting
FDR: false discovery rate
CSSPN: condition-specific shortest-path network
SCIA: self-contained and competitive incorporated analysis.
