# Supplementary material for: SCIA: A Novel Gene Set Analysis Applicable to Data With Different Characteristics
Source: Front Genet. 2019 Jun 25;10:598. doi: 10.3389/fgene.2019.00598 (PMC6603225; doi:10.3389/fgene.2019.00598)
Supplement: Supplementary file 1 [file Table_1.DOC]

Supplementary Material

# Supplementary Data Scenarios

## Scenario 1

Because one of the main advantages of self-contained methods was powerful with datasets containing relatively high inter-gene correlations, we set
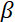
 scores as 0, 0.31, 0.65, 1.5 in Scenario 1, to make sure the Pearson Correlation Coefficients equal to 0, 0.3, 0.6, and 0.9. FDRs of the three methods (SCIA, NES statistic, and ROAST) was calculated under the condition of
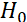
 hypothesis:
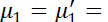
1, while the condition of
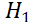
 hypothesis:
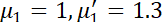
was used to calculate the sensitivities.

## Scenario 2

In Scenario 2, we explored the FDR and sensitivity of SCIA considering other DEGs outside the given pathway. Because the utilization of background gene information was one of the greatest advantages of competitive methods, we first compared the FDRs and sensitivities between SCIA and two competitive methods (CAMERA, and GSEA) under a background network. The length of background pathway
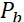
 and
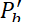
was set as
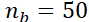
, and the targeting pathway
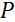
 and
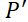
 was set as
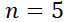
. All the rest parameter of
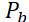
 and
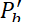
is set as the default parameters of
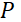
and
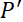
. The sample size
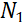
 and
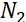
 was set as 200. To establish a CSSPN (see method),
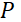
was inserted into the middle of
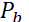
between
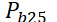
and
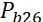
, so did
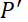
and
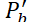
. Then, the FDR was calculated under the condition of
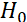
 hypothesis:
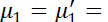
1, while the condition of
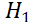
 hypothesis:
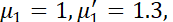
 was used to calculate the testing power. Considering the common deficiency of competitive methods,
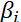
 and
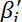
 was set as scenario 1. Specially, to prove that the FDR of SCIA was not affected by DEGs outside the given pathway, we added ten percent of DEGs randomly selected in background pathway
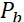
. All the selected genes
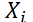
were multiplied by 1.3 after
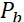
 has been generated.

## Scenario 3

In Scenario 3, we simulated the condition that there are extra DEGs (20% of the pathway) overlapping the given pathway. These DEGs, which was selected in
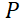
 and directly connected to the background pathway
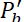
, were multiplied by 1.3. The remaining conditions were the same as Scenario 2, except the regression coefficient between genes was set as
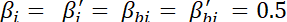
, under which condition all the three powers of different methods were comparable with similar FDRs. Specially, different percentages (0%~60%) of random DEGs in the background pathway
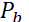
and
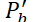
were set to show the peculiar competitive procedure of SCIA.

## Scenario 4

Through Scenario 4, we explored the sensitivity under edge changes and the influence of different sample sizes to SCIA, simultaneously. The
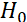
 hypothesis of Scenario 4 is the same as Scenario 1. In
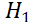
 hypothesis, the regression coefficient of
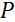
 and
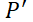
is set as
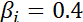
*,*
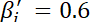
, respectively. To emphasize the role of edge changes, the mean of two experimental conditions is balanced, so the residual error term is set as
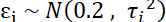
 instead of
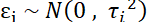
. Besides, four different sample sizes are set from 10 to 100.

# The default parameter settings of other four methods

As a comparison, we analyzed the same data using two powerful self-contained approaches: ROAST (Wu, et al., 2010; Wu and Smyth, 2012), the Node and Edge Simultaneous testing method NES (Yuan, et al., 2016), and two competitive approaches: CAMERA (Ritchie, et al., 2015; Wu and Smyth, 2012), GSEA (Subramanian, et al., 2005) under suitable conditions. In ROAST, the method is choosing as msq, and in CAMERA, the VIF is not given in advance. In GSEA, the log-FC (Fold Change) scores in different experimental conditions are used as the input ranking matrix (Zyla, et al., 2017), and the permutation time is 1000. “B-H” method (Benjamini and Hochberg, 1995) is used in all the procedures needing a p-value adjustment with the threshold as 0.05.

# Supplementary Tables

**Supplementary Table 1.** Results of KEGG pathway analysis with GSEA and the dataset of GEO

| KEGG pathway | Adjusted *p*-value |
| --- | --- |
| Endocytosis | 0.001264 |
| Epstein-Barr virus infection | 0.001297 |
| Protein processing in endoplasmic reticulum | 0.001300 |
| RNA transport | 0.001314 |
| Ubiquitin mediated proteolysis | 0.001346 |
| Ribosome | 0.001350 |
| Platelet activation | 0.001355 |
| Hepatitis C | 0.001357 |
| TNF signaling pathway | 0.001361 |
| Spliceosome | 0.001366 |
| Lysosome | 0.001368 |
| Oxidative phosphorylation | 0.001372 |
| IL-17 signaling pathway | 0.001391 |
| mRNA surveillance pathway | 0.001416 |
| EGFR tyrosine kinase inhibitor resistance | 0.001429 |
| Complement and coagulation cascades | 0.001429 |
| Proteasome | 0.001534 |
| Protein export | 0.001692 |
| Non-homologous end-joining | 0.001733 |
| Kaposi's sarcoma-associated herpesvirus infection | 0.002577 |
| Herpes simplex infection | 0.002587 |
| Measles | 0.002740 |
| Staphylococcus aureus infection | 0.003063 |
| Olfactory transduction | 0.003704 |
| Insulin signaling pathway | 0.003947 |
| Neuroactive ligand-receptor interaction | 0.004878 |
| Proximal tubule bicarbonate reclamation | 0.005038 |
| Thermogenesis | 0.005229 |
| Ribosome biogenesis in eukaryotes | 0.005806 |
| Vasopressin-regulated water reabsorption | 0.006192 |
| Insulin resistance | 0.006784 |
| Epithelial cell signaling in Helicobacter pylori infection | 0.007257 |
| Legionellosis | 0.007669 |
| SNARE interactions in vesicular transport | 0.007949 |
| Fat digestion and absorption | 0.008065 |
| Nicotine addiction | 0.008475 |
| Calcium signaling pathway | 0.008889 |
| Huntington's disease | 0.009021 |
| N-Glycan biosynthesis | 0.009146 |
| Mucin type O-glycan biosynthesis | 0.009539 |
| Non-alcoholic fatty liver disease (NAFLD) | 0.010417 |
| Dilated cardiomyopathy (DCM) | 0.010638 |
| Protein digestion and absorption | 0.010753 |
| RNA polymerase | 0.012800 |
| Hypertrophic cardiomyopathy (HCM) | 0.013378 |
| ECM-receptor interaction | 0.013937 |

**Supplementary Table 2.** Results of KEGG pathway analysis with SCIA and the dataset of GEO

| KEGG pathway | Adjusted *p*- value |
| --- | --- |
| Proteoglycans in cancer | 7.94E-18 |
| Epstein-Barr virus infection | 6.03E-16 |
| Kaposi's sarcoma-associated herpesvirus infection | 2.12E-15 |
| Hepatitis B | 3.35E-13 |
| HTLV-I infection | 6.27E-13 |
| Hepatocellular carcinoma | 1.35E-11 |
| Signaling pathways regulating pluripotency of stem cells | 5.43E-11 |
| Hepatitis C | 7.16E-10 |
| Prostate cancer | 1.08E-09 |
| Pancreatic cancer | 1.10E-09 |
| Human papillomavirus infection | 1.42E-09 |
| Tuberculosis | 1.80E-09 |
| T cell receptor signaling pathway | 2.14E-09 |
| EGFR tyrosine kinase inhibitor resistance | 2.47E-09 |
| Osteoclast differentiation | 2.95E-09 |
| C-type lectin receptor signaling pathway | 3.50E-09 |
| Small cell lung cancer | 4.07E-09 |
| Influenza A | 4.73E-09 |
| Neurotrophin signaling pathway | 5.15E-09 |
| Adherens junction | 5.25E-09 |
| Th17 cell differentiation | 5.62E-09 |
| Breast cancer | 6.20E-09 |
| Chronic myeloid leukemia | 1.15E-08 |
| Non-small cell lung cancer | 1.35E-08 |
| PI3K-Akt signaling pathway | 1.72E-08 |
| Herpes simplex infection | 1.75E-08 |
| Viral carcinogenesis | 1.87E-08 |
| Endometrial cancer | 2.23E-08 |
| Cellular senescence | 2.89E-08 |
| Wnt signaling pathway | 3.01E-08 |
| Gastric cancer | 4.27E-08 |
| AGE-RAGE signaling pathway in diabetic complications | 7.33E-08 |
| Toxoplasmosis | 8.44E-08 |
| Epithelial cell signaling in Helicobacter pylori infection | 1.67E-07 |
| Th1 and Th2 cell differentiation | 1.68E-07 |
| Natural killer cell mediated cytotoxicity | 1.83E-07 |
| Renal cell carcinoma | 2.00E-07 |
| Prolactin signaling pathway | 2.38E-07 |
| B cell receptor signaling pathway | 2.84E-07 |
| Glioma | 2.84E-07 |
| Apoptosis | 3.24E-07 |
| ErbB signaling pathway | 3.86E-07 |
| Colorectal cancer | 4.48E-07 |
| Shigellosis | 7.51E-07 |
| Fc gamma R-mediated phagocytosis | 9.16E-07 |
| Measles | 1.04E-06 |
| Ras signaling pathway | 1.13E-06 |
| Adipocytokine signaling pathway | 1.47E-06 |
| VEGF signaling pathway | 1.98E-06 |
| Inflammatory mediator regulation of TRP channels | 2.02E-06 |
| MAPK signaling pathway | 2.10E-06 |
| Leishmaniasis | 3.17E-06 |
| Thyroid hormone signaling pathway | 3.58E-06 |
| Amyotrophic lateral sclerosis (ALS) | 3.63E-06 |
| Basal cell carcinoma | 3.90E-06 |
| FoxO signaling pathway | 3.99E-06 |
| Acute myeloid leukemia | 6.25E-06 |
| Insulin resistance | 6.62E-06 |
| Legionellosis | 7.42E-06 |
| Fc epsilon RI signaling pathway | 8.43E-06 |
| NF-kappa B signaling pathway | 8.64E-06 |
| Endocrine resistance | 9.71E-06 |
| Jak-STAT signaling pathway | 1.42E-05 |
| HIF-1 signaling pathway | 1.53E-05 |
| Platinum drug resistance | 1.70E-05 |
| Focal adhesion | 1.73E-05 |
| Chagas disease (American trypanosomiasis) | 1.90E-05 |
| Bacterial invasion of epithelial cells | 1.94E-05 |
| Pertussis | 2.52E-05 |
| Hippo signaling pathway | 2.89E-05 |
| Mitophagy - animal | 3.43E-05 |
| IL-17 signaling pathway | 3.52E-05 |
| GnRH signaling pathway | 3.52E-05 |
| Pathogenic Escherichia coli infection | 5.16E-05 |
| RIG-I-like receptor signaling pathway | 6.62E-05 |
| Melanogenesis | 8.04E-05 |
| Salmonella infection | 8.11E-05 |
| Melanoma | 8.46E-05 |
| Sphingolipid signaling pathway | 8.98E-05 |
| Endocytosis | 9.37E-05 |
| Rap1 signaling pathway | 9.37E-05 |
| Regulation of actin cytoskeleton | 0.000106 |
| Toll-like receptor signaling pathway | 0.000107 |
| Insulin signaling pathway | 0.000110 |
| Fluid shear stress and atherosclerosis | 0.000129 |
| Thyroid cancer | 0.000139 |
| TNF signaling pathway | 0.000154 |
| Central carbon metabolism in cancer | 0.000197 |
| Leukocyte transendothelial migration | 0.000219 |
| Chemokine signaling pathway | 0.000259 |
| Bladder cancer | 0.000273 |
| NOD-like receptor signaling pathway | 0.000286 |
| mTOR signaling pathway | 0.000309 |
| TGF-beta signaling pathway | 0.000312 |
| Oocyte meiosis | 0.000563 |
| Phospholipase D signaling pathway | 0.000744 |
| Transcriptional misregulation in cancer | 0.000843 |
| Relaxin signaling pathway | 0.000860 |
| Amoebiasis | 0.000912 |
| Long-term potentiation | 0.001218 |
| p53 signaling pathway | 0.001343 |
| Axon guidance | 0.001350 |
| Glucagon signaling pathway | 0.001571 |
| cAMP signaling pathway | 0.001590 |
| Platelet activation | 0.001816 |
| Gap junction | 0.001862 |
| Cell cycle | 0.001938 |
| Parathyroid hormone synthesis, secretion and action | 0.001952 |
| Longevity regulating pathway | 0.002015 |
| Autophagy - animal | 0.002495 |
| Non-alcoholic fatty liver disease (NAFLD) | 0.002781 |
| Oxytocin signaling pathway | 0.003460 |
| Endocrine and other factor-regulated calcium reabsorption | 0.003539 |
| Choline metabolism in cancer | 0.004170 |
| Prion diseases | 0.004688 |
| Adrenergic signaling in cardiomyocytes | 0.006144 |
| Tight junction | 0.007979 |
| Staphylococcus aureus infection | 0.008440 |
| Phagosome | 0.009113 |
| Cushing's syndrome | 0.010005 |
| Estrogen signaling pathway | 0.011948 |
| Progesterone-mediated oocyte maturation | 0.013410 |
| MicroRNAs in cancer | 0.014641 |
| Cytosolic DNA-sensing pathway | 0.015802 |
| Inflammatory bowel disease (IBD) | 0.016960 |
| Base excision repair | 0.019947 |
| Apoptosis - multiple species | 0.019947 |
| Huntington's disease | 0.020270 |
| Amphetamine addiction | 0.020782 |
| Vibrio cholerae infection | 0.020827 |
| Dopaminergic synapse | 0.023799 |

Table S3 Results of KEGG pathway analysis with GSEA and the dataset of TCGA

**Supplementary Table 3.** Results of KEGG pathway analysis with GSEA and the dataset of TCGA

| KEGG pathway | Adjusted *p*-value |
| --- | --- |
| Alcoholism | 0.001034 |
| Estrogen signaling pathway | 0.001047 |
| Cell cycle | 0.001048 |
| Systemic lupus erythematosus | 0.001057 |
| GABAergic synapse | 0.001099 |
| Drug metabolism - other enzymes | 0.001106 |
| Chemical carcinogenesis | 0.001106 |
| Metabolism of xenobiotics by cytochrome P450 | 0.00112 |
| Retinol metabolism | 0.001138 |
| Steroid hormone biosynthesis | 0.00117 |
| Porphyrin and chlorophyll metabolism | 0.001212 |
| Nicotine addiction | 0.001212 |
| DNA replication | 0.001222 |
| Pentose and glucuronate interconversions | 0.001276 |
| Ascorbate and aldarate metabolism | 0.001289 |
| p53 signaling pathway | 0.002237 |
| Asthma | 0.00369 |
| Graft-versus-host disease | 0.003759 |
| Allograft rejection | 0.003788 |
| Intestinal immune network for IgA production | 0.004831 |
| Viral carcinogenesis | 0.005092 |
| Prion diseases | 0.005181 |
| Staphylococcus aureus infection | 0.00565 |
| Malaria | 0.005988 |
| Drug metabolism - cytochrome P450 | 0.006764 |
| Leishmaniasis | 0.007752 |
| Homologous recombination | 0.008653 |
| Neuroactive ligand-receptor interaction | 0.009045 |
| Fc epsilon RI signaling pathway | 0.009259 |
| Renin secretion | 0.009259 |
| Fanconi anemia pathway | 0.009524 |
| African trypanosomiasis | 0.009662 |
| Complement and coagulation cascades | 0.010309 |
| Mismatch repair | 0.01039 |
| Oocyte meiosis | 0.010482 |
| Hematopoietic cell lineage | 0.010989 |
| Biosynthesis of amino acids | 0.011173 |
| Phototransduction | 0.01148 |
| Ribosome | 0.01153 |
| Th17 cell differentiation | 0.011905 |

**Supplementary Table 4.** Results of KEGG pathway analysis with SCIA and the dataset of TCGA

| KEGG pathway | Adjusted *p*-value |
| --- | --- |
| Cell cycle | 6.45E-12 |
| Viral carcinogenesis | 7.50E-09 |
| Proteoglycans in cancer | 4.04E-08 |
| Pathogenic Escherichia coli infection | 4.47E-06 |
| Hepatitis B | 6.41E-06 |
| Kaposi's sarcoma-associated herpesvirus infection | 6.80E-06 |
| Thyroid hormone signaling pathway | 1.60E-05 |
| Apelin signaling pathway | 1.81E-05 |
| Mitophagy - animal | 1.84E-05 |
| Cellular senescence | 2.16E-05 |
| p53 signaling pathway | 2.66E-05 |
| Colorectal cancer | 3.01E-05 |
| Glioma | 3.79E-05 |
| Bladder cancer | 4.02E-05 |
| Oxytocin signaling pathway | 5.83E-05 |
| FoxO signaling pathway | 5.86E-05 |
| Small cell lung cancer | 5.95E-05 |
| Nucleotide excision repair | 9.99E-05 |
| Herpes simplex infection | 0.000107 |
| Vascular smooth muscle contraction | 0.00012 |
| TGF-beta signaling pathway | 0.000144 |
| Long-term potentiation | 0.000159 |
| Thyroid cancer | 0.000196 |
| cAMP signaling pathway | 0.00022 |
| Focal adhesion | 0.000232 |
| Epstein-Barr virus infection | 0.000285 |
| HTLV-I infection | 0.00031 |
| Pancreatic cancer | 0.00035 |
| Chronic myeloid leukemia | 0.000383 |
| Hepatocellular carcinoma | 0.000569 |
| Melanogenesis | 0.000581 |
| Non-small cell lung cancer | 0.00085 |
| Renal cell carcinoma | 0.001111 |
| Salivary secretion | 0.001192 |
| Ubiquitin mediated proteolysis | 0.001389 |
| Adherens junction | 0.00143 |
| Melanoma | 0.00143 |
| Bacterial invasion of epithelial cells | 0.001679 |
| Huntington's disease | 0.001908 |
| Tight junction | 0.002144 |
| AGE-RAGE signaling pathway in diabetic complications | 0.002202 |
| Alzheimer's disease | 0.002246 |
| Endometrial cancer | 0.002274 |
| Glucagon signaling pathway | 0.002825 |
| Autophagy - animal | 0.00309 |
| Hippo signaling pathway | 0.003302 |
| Human papillomavirus infection | 0.003315 |
| Transcriptional misregulation in cancer | 0.004298 |
| MicroRNAs in cancer | 0.00442 |
| Gap junction | 0.004503 |
| Endocrine and other factor-regulated calcium reabsorption | 0.004675 |
| Amphetamine addiction | 0.005072 |
| Adipocytokine signaling pathway | 0.005449 |
| RNA transport | 0.006895 |
| Endocrine resistance | 0.007233 |
| Breast cancer | 0.00764 |
| Prostate cancer | 0.007645 |
| SNARE interactions in vesicular transport | 0.007887 |
| Platelet activation | 0.008243 |
| Non-alcoholic fatty liver disease (NAFLD) | 0.00832 |
| Gastric cancer | 0.00832 |
| HIF-1 signaling pathway | 0.008986 |
| Rap1 signaling pathway | 0.009102 |
| Long-term depression | 0.013044 |

**Supplementary Table 5.** Most of the genes in the CSSPN of SCIA have been reported related to lung cancer

| Gene | Sub-type of lung cancer that the gene have been reported related to |
| --- | --- |
| CCDC85B |  |
| KPNA2 | Non-small lung cancer cell (NSCLC) |
| BRCA1 | NSCLC |
| CDK1 |  |
| PPP1CA | Small cell lung cancers (SCLC) and NSCLC |
| CDK9 | Human A549 cell line (lung carcinoma) |
| CASP3 | NSCLC |
| CREBBP | SCLC |
| EP300 |  |
| MELK | SCLC |
| CSNK2A1 | NSCLC |
| MAPK1 |  |
| TP53 | Lung adenocarcinoma |
| GSK3B | Human A549 cells line |
| RPA2 |  |
| SMAD4 | NSCLC |
| SETDB1 | NSCLC |
| YWHAG | NSCLC |
| SF3B1 | Lung adenocarcinoma |
| SMAD5 | Lung adenocarcinoma |
| ESR1 | NSCLC |
| ATM | SCLC |
| E2F1 | SCLC |
| EWSR1 |  |
| NDRG1 | NSCLC |
| CTNNB1 | SCLC and NSCLC |
| PTN | NSCLC |
| TRAF1 | NSCLC |
| XRCC6 | lung adenocarcinoma |
| CDCA3 | NSCLC |
| HDAC1 | Lung squamous cell carcinoma |
| ABL1 | NSCLC |
| PTPN6 |  |
| PPP2CA |  |
| PLSCR1 |  |
| KRTAP4-12 |  |
| FLNA | Lung adenocarcinoma |
| DNAJA3 |  |
| AR |  |
| SGOL1 |  |
| KAT5 |  |

# Reference

Benjamini, Y. and Hochberg, Y. (1995) Controlling the talse discovery rate - a practical and powerful approach to multiple testing. J. R. Stat. Soc. B, 57, 289-300.

Ritchie, M.E. et al. (2015) limma powers differential expression analyses for RNA-sequencing and microarray studies. Nucleic Acids Res., 43, e47.

Subramanian, A. et al. (2005) Gene set enrichment analysis: a knowledge-based approach for interpreting genome-wide expression profiles. Proc. Natl. Acad. Sci. USA, 102, 15545-15550.

Wu, D. et al. (2010) ROAST: rotation gene set tests for complex microarray experiments. Bioinformatics, 26, 2176-2182.

Wu, D. and Smyth, G.K. (2012) Camera: a competitive gene set test accounting for inter-gene correlation. Nucleic Acids Res., 40, e133.

Yuan, Z. et al. (2016) A novel chi-square statistic for detecting group differences between pathways in systems epidemiology. Stat. Med., 35, 5512-5524.

Zyla, J. et al. (2017) Ranking metrics in gene set enrichment analysis: do they matter? BMC Bioinformatics, 18, 256.
